# Nanostructural Modification of PEDOT:PSS for High Charge Carrier Collection in Hybrid Frontal Interface of Solar Cells

**DOI:** 10.3390/polym11061034

**Published:** 2019-06-11

**Authors:** Antonio J. Olivares, Ismael Cosme, Maria Elena Sanchez-Vergara, Svetlana Mansurova, Julio C. Carrillo, Hiram E. Martinez, Adrian Itzmoyotl

**Affiliations:** 1 National Institute of Astrophysics, Optics and Electronics (INAOE), Luis Enrique Erro # 1, Tonantzintla, Puebla, C.P. 72840, Mexico; adj.olivares@inaoep.mx (A.J.O.); smansur@inaoep.mx (S.M.); hiram@inaoep.mx (H.E.M.); aitzmo@inaoep.mx (A.I.); 2 Consejo Nacional de Ciencia y tecnología—INAOE, Luis Enrique Erro # 1, Tonantzintla, Puebla C.P. 72840, Mexico; 3 Facultad de Ingeniería, Universidad Anáhuac México, Av. Universidad Anáhuac 46, Col. Lomas Anáhuac, Huixquilucan C.P. 52786, Mexico; elena.sanchez@anahuac.mx; 4 UAM Reynosa—Rodhe, Universidad Autónoma de Tamaulipas, Carr. Reynosa-San Fernando S/N, Reynosa, Tamaulipas 88779, Mexico; julioccs.88@gmail.com

**Keywords:** PEDOT:PSS, conductive polymer, hybrid interface, silicon

## Abstract

In this work, we propose poly(3,4-ethylenedioxythiophene)-poly(styrenesulfonate) (PEDOT:PSS) material to form a hybrid heterojunction with amorphous silicon-based materials for high charge carrier collection at the frontal interface of solar cells. The nanostructural characteristics of PEDOT:PSS layers were modified using post-treatment techniques via isopropyl alcohol (IPA). Atomic force microscopy (AFM), Fourier-transform infrared (FTIR), and Raman spectroscopy demonstrated conformational changes and nanostructural reorganization in the surface of the polymer in order to tailor hybrid interface to be used in the heterojunctions of inorganic solar cells. To prove this concept, hybrid polymer/amorphous silicon solar cells were fabricated. The hybrid PEDOT:PSS/buffer/a-Si:H heterojunction demonstrated high transmittance, reduction of electron diffusion, and enhancement of the internal electric field. Although the structure was a planar superstrate-type configuration and the PEDOT:PSS layer was exposed to glow discharge, the hybrid solar cell reached high efficiency compared to that in similar hybrid solar cells with substrate-type configuration and that in textured well-optimized amorphous silicon solar cells fabricated at low temperature. Thus, we demonstrate that PEDOT:PSS is fully tailored and compatible material with plasma processes and can be a substitute for inorganic p-type layers in inorganic solar cells and related devices with improvement of performance and simplification of fabrication process.

## 1. Introduction

Organic materials for photovoltaic (PV) applications have been in focus during the last decade. One important advantage of these materials is their solution-type processing that allows deposition at room temperature and atmospheric pressures. Recently, these materials have also attracted attention as an alternative of common n-type and p-type inorganic materials to simplify the formation of p-/i-/n-junctions in solar cells [1,2,3]. However, inorganic materials are still the most widely used as an alternative to form these heterojunctions in the frontal interface. For example, doped, nanocrystalline SiO_x_:H layers were proposed by Fang et. al. in 2018 [4] in order to improve performance characteristics in amorphous silicon solar cells, but these configurations still involve complex optimizations at the n/i or i/p interfaces similar to those in the interfaces based on doped-SiC:H layers. Some preliminary attempts to use organic materials in hybrid n/i and i/p heterojunctions with amorphous silicon have been proposed by the National Renewable Energy Laboratory in 2008 [5] and L’Ecole Polytechnique, France in 2007 [6]. However, performance characteristics were limited (*J*_sc_ < 4 mA/cm^2^) by characteristics of the polymers and their poor optimization for use in a hybrid heterojunction interface with amorphous silicon.

Highly conductive polymer poly(3,4-ethylenedioxythiophene):poly(4-styrenesulfonate) (PEDOT:PSS) has proved to be an innovative material from a new perspective for application in silicon photovoltaic (PV) technology [2,7,8]. In hybrid crystalline silicon (c-Si) solar cells, PEDOT:PSS has been demonstrated to be a very efficient electron blocker, acting as “ideal” barrier [9,10,11] or passivation material [12] in heterojunction intrinsic thin film (HIT) solar cells, acting as a substitute of p-type material. PEDOT:PSS thin films have also shown potential to be used with amorphous silicon as a p-type window layer in PV devices [13,14,15] due to their large bandgap (~1.6 eV) and high work function (~5 eV). Moreover, the p-type polymer layer has the potential to simplify the fabrication of the frontal interfaces.

The first proof of concept of an amorphous silicon solar cell using a PEDOT:PSS/(i) a-Si:H heterojunction in superstrate-type configuration was presented by Williams et al. in 2005 [16]. This device showed a power conversion efficiency (PCE) as large as 2.1%. Then, Jung et al. (2013) [17] and Peng et al. (2014) [3] improved the PCE of these configurations to values of 5.49% and 4.78%, respectively, via manipulation of the hybrid interface. However, it is widely believed that the performance parameters of superstrate-type devices are limited by the degradation of PEDOT:PSS due to exposure to high temperature (>200 °C) and ion bombardment during the plasma deposition of subsequent layers. Thus, Lee et al., in 2018 [13], fabricated a substrate-type solar structure in order to avoid exposure of the PEDOT:PSS layer to plasma-enhanced chemical vapor deposition (PECVD). As a result, the performance characteristics of the substrate-type configuration were considerably higher than those in superstrate-type configuration. Finally, improving the wettability of PEDOT:PSS on a-Si:H film and adding a p-type a-Si:H buffer layer, Lee et al. [13] increased the PCE of these structures on glass substrate from 3.10% to 7.40%.

In the field of pure organic solar cell technology, it is well known that the characteristics of PEDOT:PSS should be modified by applying physical or chemical postdeposition treatments in order to tailor characteristics according to the function of the conducting polymer [18,19,20]. For example, the dark conductivity of PEDOT:PSS films can be modified by orders of magnitude via solvent treatment [21,22]. Using this concept, our group in 2017 [23] has previously reported the influence of post-treatment on PEDOT:PSS/a-Si:H interface in solar cells. It has been demonstrated that open-circuit voltage (*V*_oc_), short-circuit current (*J*_sc_), and parasitic resistance and fill factor (FF) can be improved by using isopropyl alcohol (IPA). In this case, the best device exhibited a *J*_sc_ as a large as 15.29 mA/cm^2^ and a PCE = 3.40% [24]. We demonstrated that, like in organic solar cells, PEDOT:PSS films also require optimization via treatments for application in organic–inorganic heterojunctions.

The aim of this work is to demonstrate the improvements and advantages of using PEDOT:PSS polymer to form a hybrid heterojunction with amorphous silicon in the frontal interface of solar cells. The nanostructural, morphological and electronic characteristics of the PEDOT:PSS layers were tailored using different post-treatment techniques via isopropyl alcohol (IPA). To demonstrate this idea, we have fabricated superstrate-type amorphous silicon solar cells with different configurations. Until now, it was believed that PEDOT:PSS applications in hybrid structures were limited to substrate-type configurations [13] in order to avoid degradation of the polymer due to plasma exposure. However, we have shown that PEDOT:PSS is fully compatible with plasma processes and can be tailored in the bulk and surface characteristics to be a substitute of inorganic p-type layers in superstrate-type solar cells with an improvement of performance characteristics and a simplification of the fabrication process.

## 2. Materials and Methods

**Figure 1:**
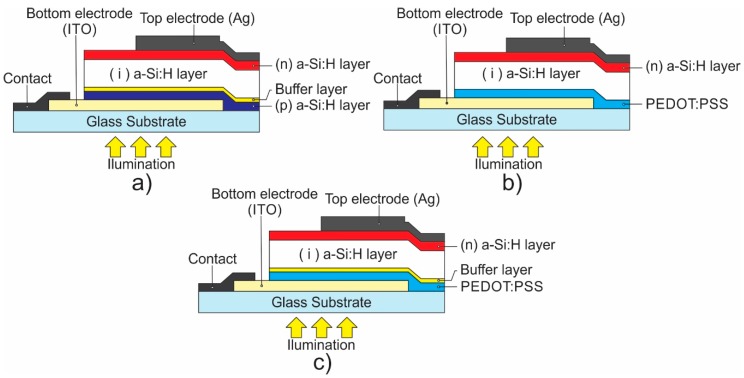
Schematics of (**a**) photovoltaic structure based on amorphous silicon (a-Si:H reference sample), (**b**) superstrate-type hybrid structure (H1) with PEDOT:PSS/a-SiH heterojunction in frontal interface, and (**c**) superstrate-type hybrid structure (H2) with PEDOT:PSS/buffer/a-SiH heterojunction in frontal interface.

Figure 1 shows the diagrams of the different configurations for the photovoltaic structures fabricated in this work. A superstrate pin-type a-Si:H solar cell was fabricated as a reference sample (Figure 1a) according to the following stages and conditions; the structure was fabricated on glass substrates coated with a 160-nm-thick tin-doped In_2_O_3_ (ITO) film with an sheet resistance of 14 Ω/sq, purchased from Precision Glass & Optics (PG&O^®^) (Santa Ana, CA, USA). The ITO electrode was defined by hydrochloric acid etching and cleaned using acetone and isopropyl alcohol ultrasonic baths, sequentially. The a-Si:H-based layers were deposited using a cluster tool from MVSystem Inc. (Denver, CO, USA). This installation is a semi-industrial commercial system for fabrication of multilayered optoelectronic structures based on different materials deposited by plasma (in three radio frequency (RF) PECVD chambers: intrinsic, n-type, and p-type processes). The inorganic films were deposited from silane SiH_4_(10%)/H_2_, methane CH_4_, phosphine PH_3_(1%)/H_2_ and diborane B_2_H_6_(1%)/H_2_ gas sources purchased from MATHESON (Montgomeryville, Pensilvania, USA) with 5N purity. The 15-nm-thick p-type a-SiC:H:B layer was deposited from SiH_4_, CH_4_, and B_2_H_6_ mixtures at flow rates of 30 sccm, 7 sccm, and 3 sccm, respectively, at pressure *P* = 0.67 Torr. A buffer layer was deposited to form a gradient a-SiC:H/a-Si:H interface between the p-type and the active layer. The active layer was deposited from SiH_4_ mixtures at a flow rate of 10 sccm to form a 300-nm-thick a-Si:H film. Then, a 10 nm-thick Si:H n-type layer was deposited from SiH_4_ and PH_3_ mixtures at a flow rate of 30 sccm and 0.3 sccm, respectively. Intrinsic and n-type layers we deposited at pressure P = 0.55 Torr. During all deposition processes, the deposition temperature was kept constant at T_d_ = 160 °C, and the power was fixed at 3 W. Finally, a top Ag contact was deposited using sputtering through a metal shadow mask with an area of 0.1 cm^2^.

**Figure 2:**
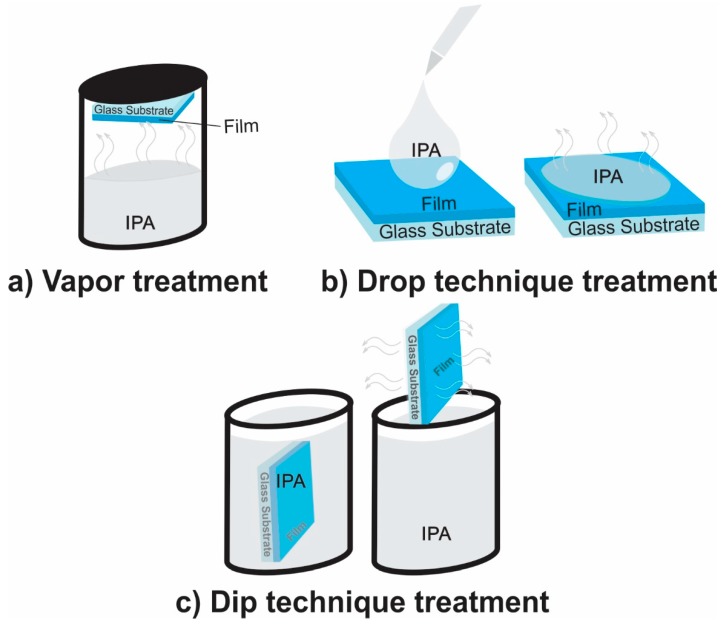
Schematic illustration of different solvents treatments used in this work: (**a**) vapor, (**b**) drop, and (**c**) dip techniques.

Different hybrid structures were fabricated, maintaining a sequence of operations and their conditions similar to those described above for the reference sample, with changes in the frontal interface, as is indicated in each configuration (Figure 1). The PEDOT:PSS blend mixture (1:6 weight ratio) was prepared from components purchased from Ossila Ltd. (Sheffield, UK). This mixture was deposited by spin-coating at a rotation speed of 2000 rpm to obtain films with thickness around ~45 nm. Finally, the PEDOT:PSS films used in the structures were post-treated via IPA vapor exposure (Figure 2a), IPA dropped on the surface (Figure 2b), or dipping into the IPA solution (Figure 2c) during 20 min (H1 and H2 samples) and 40 min (H6 sample)**.** The current density–voltage characteristics *J*(U) of the solar cells were measured using a 6517A electrometer from Keithley (Cleveland, OH, USA) under standard AM1.5 illumination provided by a solar simulator Oriel 2A from Newport Corp Inc. (Irvine, CA, USA). External quantum efficiency (EQE) was obtained using a system with a xenon light source lamp, chopper, a monochromator, a lock-in amplifier from Stanford research system (Sunnyvale, CA, USA) and a Si reference detector provided by Newport corporation.

Morphological characteristics of PEDOT:PSS films have been investigated via atomic force microscopy (AFM) using an NTEGRA platform from NT-MDT Inc (Liestal, Switzerland). The optical transmittances of the films on glass substrates were measured using an MProbe 20 UV–Vis spectrometer from SemiconSoft Inc (Southborough, MA, USA). Work function was measured using an experimental setup with a Kelvin probe from Besocke Delta Phi (Jülich, Germany) with a probe diameter of ~3 mm in air and at room temperature. Resistivity was characterized by applying the four-point probe method using a Keithley 6517A electrometer. Raman spectra were measured using an AFM-Raman of Ntegra Spectra systems from NT-MDT Inc. with a wavelength of the excitation laser of 532 nm. Finally, FTIR analysis was carried out in PEDOT:PSS films on silicon wafers using a Nicolet iS5-FT spectrometer from Thermo Scientific Nicolet Inc.

## 3. Results and Discussion

**Figure 3:**
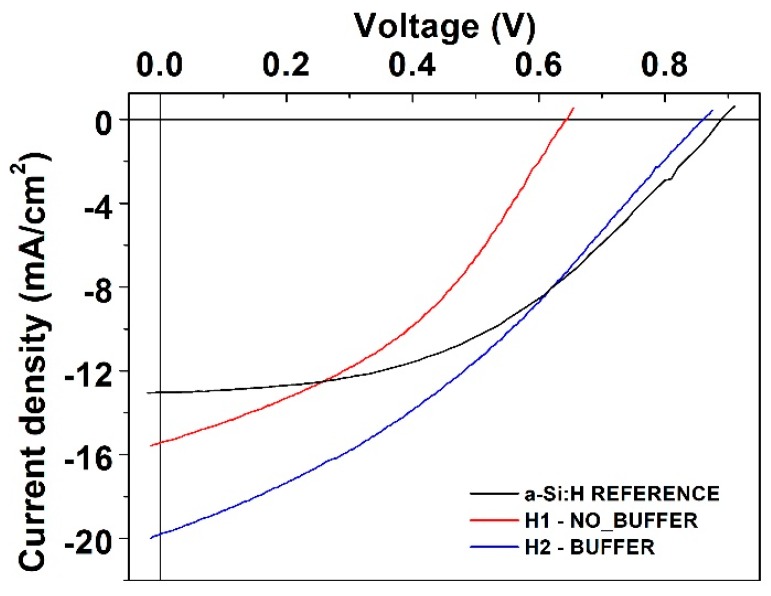
Performance parameters of hybrid photovoltaic structures fabricated with pristine and post-treated PEDOT:PSS films.

Figure 1 shows the schematic of the fabricated structures; in previous work, we reported a glass substrate ITO/PEDOT:PSS/(i) a-Si:H/ (n) a-Si:H structure [24], labeled in this work as the H1-NO_BUFFER configuration (Figure 1b). Now, we have added an optimized buffer layer that consists of a very thin graded amorphous silicon carbide (a-SiC:H/a-Si:H) layer between the conductive polymer and a a-Si:H absorber layer to form a GLASS SUBSTRATE/ITO/PEDOT:PSS/buffer layer/(i) a-Si:H/n a-Si:H structure labeled as the H2-BUFFER configuration (Figure 1c). Figure 3 and Table 1 present the density–voltage (*J*(U)) characteristics, under AM1.5 illumination of the H1, H2, and amorphous reference structures. It is observed that the addition of a buffer layer in the hybrid configuration (H2-BUFFER) results in increases of *V*_oc_ from 640 mV to 860 mV and *J*_sc_ from 15.43 mA/cm^2^ to 19.8 mA/cm^2^ compared to that in the sample without the buffer layer (H1-NO_BUFFER structure).

**Figure 4:**
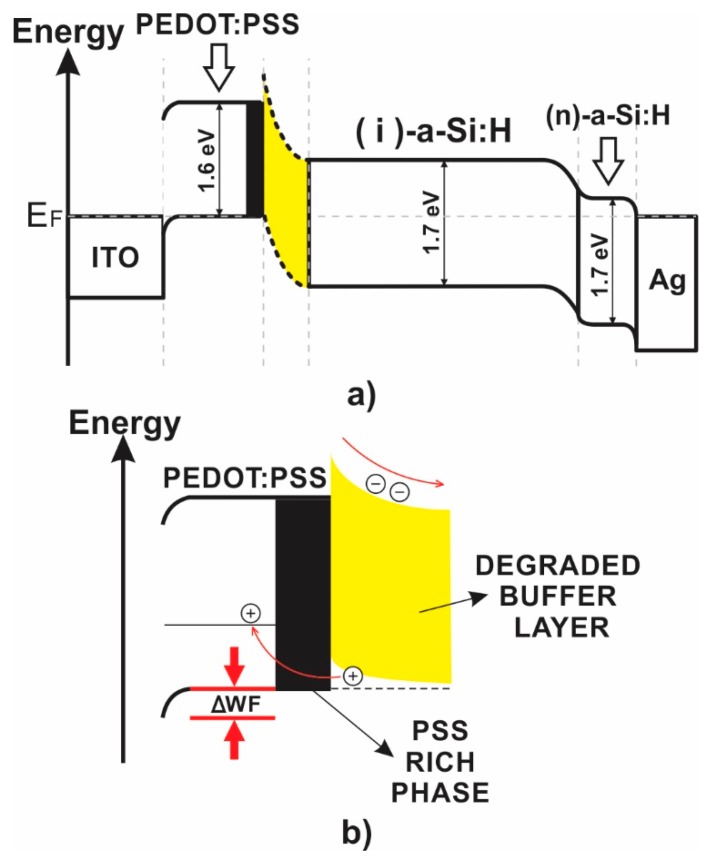
(**a**) Energy level band diagram in thermal equilibrium for a hybrid structure and (**b**) schematic representation of the electron-blocking mechanism with the buffer layer (SIC:H/Si:H) by interfacial wide energy gap PSS [26].

The hypothetical energetic diagram for electrons in thermal equilibrium for the H2-BUFFER structure is presented in Figure 4 as a guide for discussion. The values of work functions, optical gaps, electron affinities, and detailed information used for the construction of this diagram are discussed in a previous work [24]. In pure a-Si:H cells, it is well known that a-SiC:H:B/buffer layer systems are used as p-type layer in the frontal interface to increase “built-in” electric field and doped-layer transparency [25]. In the case of hybrid configurations using PEDOT:PSS, buffer layers have the same effect. The similarity between the optical gaps of amorphous silicon (*E*_g_ ~1.7 eV) and PEDOT:PSS (*E*_g_ ~1.6 eV) results in low *V*_oc_ ~640 mV in the H1-NO_BUFFER structure compared to those in structures with buffer layer (H2-BUFFER and reference structures). It is important to note that the use of an intrinsic SiC:H buffer layer in the frontal interface in hybrid configuration improves the open-circuit voltage to 840 mV, which is very close to that in pure a-Si:H configuration with *V*_oc_ = 840 mV. The improvement in *V*_oc_ can be explained as consequence of the creation of a potential barrier in the conduction band at the PEDOT:PSS/SiC:H-Si:H interface, improving the built-in potential, and a reduction of the electron diffusion into the PEDOT:PSS film (Figure 4b).

**Figure 5:**
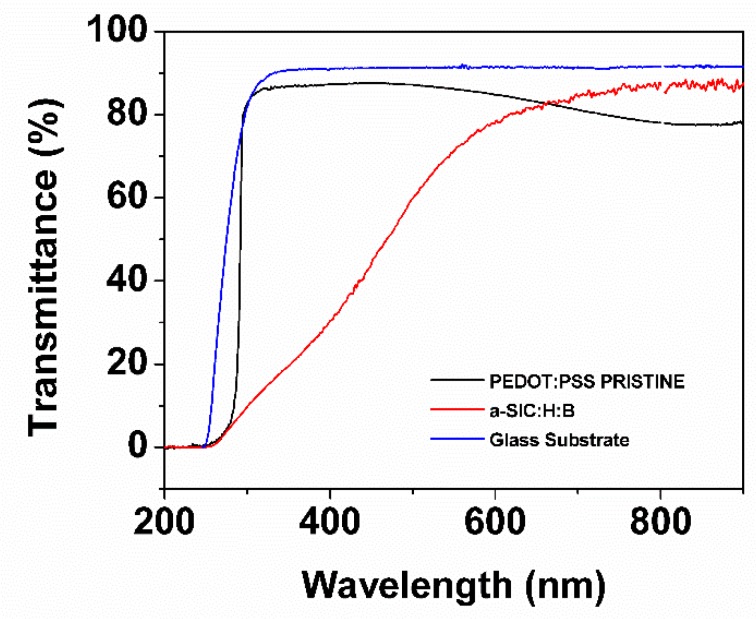
Optical transmittance spectra of an amorphous silicon carbine film doped with boron (a-SiC:H:B), PEDOT:PSS pristine film, and glass substrate.

It is also observed that the hybrid configurations–the H1 and H2 structures–have higher values of *J*_sc_ than that obtained in the a-Si:H reference structure. The higher *J*_sc_ in the H1-NO_BUFFER structure compared to that in the reference structure may relate to the relatively high optical transmission of PEDOT:PSS. Figure 5 shows the optical transmittance spectra *T*(λ) of the PEDOT:PSS and the a-SiC:H:B layers deposited on glass substrate. Glass/PEDOT:PSS system shows a remarkable transmittance with an average value of *T*_avg_ = 85% in the wavelength range from 300 to 750 nm. This contrasts with the transmittance of the Glass/a-SiC:H:B system, in which the average value is *T*_avg_ = 59%. This represents an increase of ~25% of light reaching the absorber layer in the hybrid configurations. However, the H2-BUFFER configuration showed the highest *J*_sc_ with value of 19.8 mA/cm^2^. This can be attributed to various factors such as the relatively better transmission of PEDOT:PSS, a better hole collection at the frontal interface improved by the buffer layer and a good alignment between valence band conduction of silicon and PEDOT:PSS work function.

Table 1 shows the fill factor (FF) and parasitic resistance values of the H1, H2, and reference structures. Hybrid structures have a drastically decrease in shunt resistance, compared to that in the a-Si:H reference structure from R_sh_ ~1.4 kΩ cm^2^ to 100 Ω cm^2^. However, the serial resistance related to the hybrid structures showed similar values compared to that in the reference structure. Consequently, the fill factor is affected by the increase of R_sh_ and this is lower in the hybrid structures. These differences between the hybrid configurations and the a-Si:H configuration may be related to the use of the PEDOT:PSS layer in the frontal interface. Fill factor and parasitic resistances are very complex parameters involved in the solar cell performance, and these can be affected by several aspects and mechanisms in inorganic and organic configurations. In this case, the nonoptimized hybrid heterojunction and the acidity of PEDOT:PSS [27], affecting ITO and a-Si:H layer, may explain the abrupt reduction of shunt resistance in the hybrid configurations.

Several methods have been proposed to enhance electronic and morphological characteristics of PEDOT:PSS films via solvent post-treatments [7,28,29] in order to improve FF, parasitic resistances, and performance characteristics in organic solar cells. Following these approaches, we have demonstrated that the same idea can be used to control PEDOT:PSS film characteristics for application in hybrid PEDOT:PSS/a-SI:H interfaces [23]: an improvement of conductivity can be obtained in PEDOT:PSS films via IPA post-treatments; thus, to better understand and control PEDOT:PSS film characteristics for hybrid PEDOT:PSS/buffer/a-Si:H heterojunctions, the effects of three different IPA post-treatment techniques on the electrical, optical, and morphological characteristics of PEDOT:PSS films are studied.

**Figure 6:**
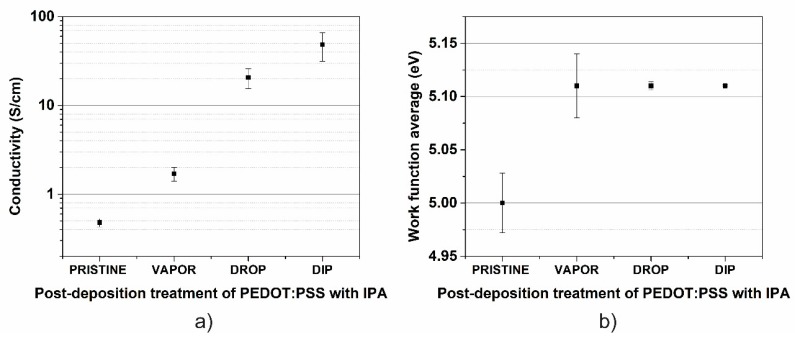
(**a**) Electrical conductivity and (**b**) work function of pristine PEDOT:PSS film and post-treated IPA PEDOT:PSS films using VAPOR, DROP, and DIP techniques.

Figure 6a shows the conductivity of pristine and post-treated PEDOT:PSS films via the vapor, drop and dip (45 min) techniques. The lowest electrical conductivity is obtained in the pristine film (*σ* = 0.48 S/cm) and increases as the aggressivity of the treatment is increased from vapor (1.7 S/cm), drop (20.7 S/cm), and dip (48.5 S/cm) techniques; a control of electronic conductivity by two orders of magnitude in PEDOT:PSS using different post-treatment techniques was achieved, similar to that in doped inorganic semiconductors, but in a vacuum-free and low-temperature process. PSS distribution and PEDOT/PSS ratio have a direct impact on charge transport for the conductive polymer [18,30]. IPA treatment removes PSS and affects coulomb interaction between PEDOT and PSS chains, modifying the internal distribution and improving the average conductivity of the film. In this case, the post-treatment dip technique allows deeper penetration of IPA to modify PSS chains into the bulk compared to the vapor or drop techniques that affect only the surface.

**Figure 7:**
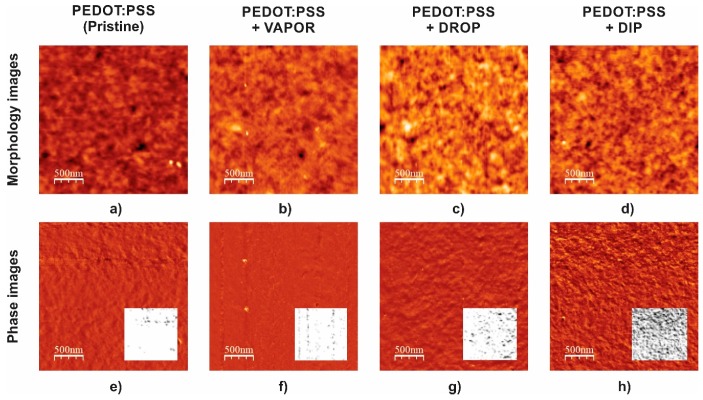
Atomic force microscopy (AFM) (morphology and phase) images of (**a**) and (**e**) pristine PEDOT:PSS film post-treated IPA PEDOT:PSS films using (**b**) and (**f**) VAPOR, (**c**) and (**g**) DROP, and (**d**) and (**h**) DIP techniques; high-contrast phase images (inset images)

Figure 6b shows the values of work function of the pristine and post-treated films. An increase in the work function with respect to the value in the pristine PEDOT:PSS film (5.0 eV) was observed in all of the post-treated samples with a value of ~5.11 eV. The mechanisms that determine PEDOT:PSS work function are usually complex and confusing, and this can be affected by PEDOT/PSS ratio, preparation and/or ambient exposure [26,31,32]. Our results suggest that the changes in work function in post-treated PEDOT:PSS films can be attributed to changes in the surface caused by enrichment of PPS chains due to segregation from the bulk. Figure 7 and Table 2 present the atomic force microscopy images and morphological analysis of the surface of the pristine and post-treated films. The surface of the pristine PEDOT:PSS film shows the lowest value of roughness *R*_q_ = 1.57 nm, and an increase with IPA post-treatment is observed to a maximum of *R*_q_ = 1.93 nm using the dip technique. The increase of surface roughness in treated PEDOT:PSS films is well reported and may occur due to redistribution of PSS and PEDOT chains [32,33,34]. To confirm this, Figure 7e–h shows the AFM phase images and their high-contrast version for the different PEDOT:PSS films. A smooth image of the surface is obtained for the pristine sample, and the degree of phase contrast clearly increases with the increase of IPA treatment aggressivity from the vapor to the dip technique. Accumulation of PSS on the surface is clearly observed in the high-contrast images as the increase of negative/dark regions (PSS-rich domains), which explains the increase of work function in the post-treated films. This confirms a clear separation and aggregation of PEDOT-rich and PSS-rich domains as the aggressivity of IPA treatment is increased. The latter can also explain the increase of conductivity in the treated PEDOT:PSS films as consequence of a self-organized nanostructure process; the PSS-enrichment of the surface will result in an accumulation of PEDOT chains in the bulk, improving channels of charge transport and increasing the conductivity.

The calculated kurtosis (Ku) and skewness (Sk) values for pristine and post-treated PEDOT:PSS films are shown in Table 2. Analysis of the surface kurtosis and skewness provides relevant information about morphology characteristics and changes related to the post-treatment. Kurtosis reveals whether the morphology of the surface is dominated by sharp (Ku > 3) or bumpy (Ku < 3) shapes [35], while skewness describes whether the morphology is pore (Sk < 0) or peak (Sk > 0) dominated [35]. Based on this, it is observed that the morphology for all samples is dominated by sharper peaks; however, sharpness and peak shapes are reduced by post-treatments, and surface morphology reaches more regularity (Sk ~ 0 and Ku ~ 3) with the increase of aggressivity of drop and dip post-treatments. Uniformity of the morphology of the surface of PEDOT:PSS films should play an important role in the formation of the frontal interface during deposition of amorphous silicon in subsequent stages.

**Figure 8:**
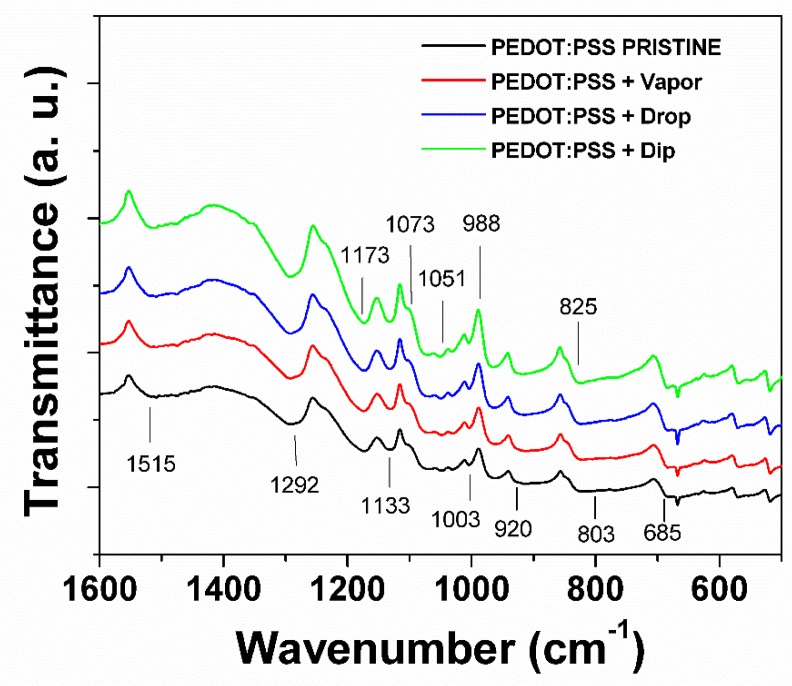
Fourier-transform infrared spectroscopy (FTIR) spectra of pristine PEDOT:PSS film post-treated IPA PEDOT:PSS films using VAPOR, DROP, and DIP techniques.

In order to better understand the morphologic changes at a molecular level caused by the post-treatment with IPA, the films were studied using FTIR and Raman spectroscopies. The chemical structure and the presence of the functional groups in the FTIR spectrum of PEDOT:PSS were observed in the pristine and post-treated films (Figure 8). The typical bands in PEDOT:PSS, such as 1515, 1292, 1173, 1133, 1073, 1051, 966, 920, 825, and 685 cm^−1^, were identified [36,37,38]. The bands near 1515 and 1292 cm^−1^ are assigned to the C=C asymmetric stretching mode and C–C inter-ring stretching mode, respectively. The C–O–C bending vibrations in the ethylenedioxy group occur at 1173, 1133, and 1051 cm^−1^, while C–S–C stretching vibrations in the thiophene ring occur at 966, 920, 825, and 685 cm^−1^. The 1190 and 1003 cm^−1^ bands are assigned to the S=O and O–S–O symmetric stretching modes in PSS, respectively. Finally, C–H angular deformation of the aromatic ring in PSS gives a band at 803 cm^−1^ [37,38]. The clear presence of these bands means that the deposition and post-treatment were carried out without degradation of the polymer. A second consideration is that the bands in the spectra of the post-treated films show a minimum displacement with respect to themselves, which confirms the chemical stability of the films. The polymerization degree of PEDOT can be evaluated from the ratio of integration of the IR bands at 825 and 685 cm^−1^; lower intensity ratio indicates a higher degree of polymerization [36]. Although the differences between ratios in the films are small, the films can be ordered by their degree of polymerization: PEDOT:PSS+DROP < PEDOT:PSS + vapor < PEDOT:PSS + dip < pristine PEDOT:PSS, indicating the highest polymerization degree in the drop post-treated PEDOT:PSS sample [36]. However, no clear trend related to the post-treatment techniques is observed. It is also important to note that the functional groups of IPA: (i) O–H stretch at 3349 cm^−1^; (ii) in-plane bend at 1309 cm^−1^; (iii) O–H wag at 655 cm^−1^; (iv) C–C–O symmetrical stretch at 817 cm^−1^; and (v) CH_3_–C–CH_3_ stretch at 952 cm^−1^, are not detected in any FTIR spectrum of the films. Thus, the small changes of polymerization degree between the pristine and post-treated films were related to the variability of the deposition process of the films rather than the treatment with IPA, and the increase of electric conductivity may be related to conformational changes rather than a modification in their chemical structure of the films.

**Figure 9:**
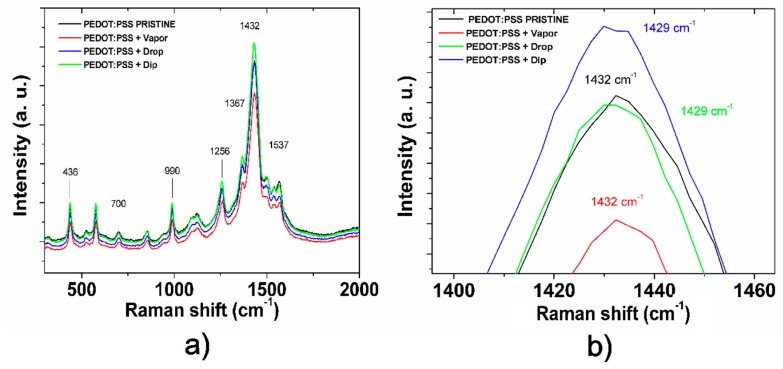
(**a**) Raman spectra of pristine PEDOT:PSS film post-treated IPA PEDOT:PSS films using VAPOR, DROP, and DIP techniques. (**b**) Close view of Raman band associated to C=C symmetrical stretching intramolecular vibration of PEDOT between 1400 and 1460 cm^−1^ (benzoid/quinoid forms).

**Figure 10:**
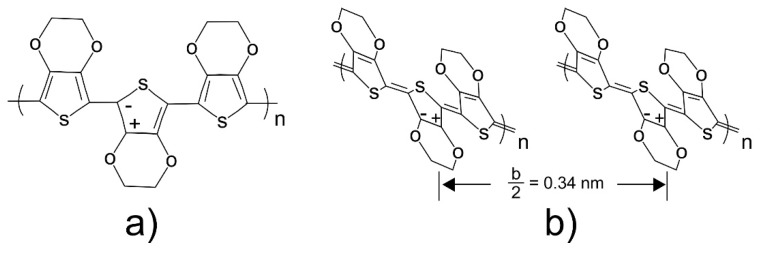
Chemical structures of the (**a**) benzoid and (**b**) quinoid forms of PEDOT.

To confirm this, the conformational changes of PEDOT in the pristine and post-treated films were studied using Raman spectroscopy (Figure 9). PEDOT has the two types of structures shown in Figure 10: (a) the benzoid structure presented in the base state and (b) quinoid structure presented in a doped state [39]. PPS associates to PEDOT through ionic bonds and acts as dopant and balancer of charge during the polymerization. The principal peaks of the chain conformation bands for PEDOT were detected in the Raman spectra as follows; C–O–C deformation at 436 cm^−1^, symmetric C–S–C deformation at 700 cm^−1^, oxyethylene ring deformation at 990 cm^−1^, and C_α_–C_α_ inter-ring stretching at 1256 cm^−1^ [36,37]. The band near 1367 cm^−1^ represents the C_β_–C_β_ stretching mode, the asymmetrical stretching vibrations are near 1537 cm^−1^ [40,41], and C_α_=C_β_ symmetric stretching vibrations are near 1434 cm^−1^. Finally, the C_α_=C_β_ symmetric stretching vibrations of the five-membered thiophene ring on PEDOT occur between 1400 and 1500 cm^−1^. Several studies on Raman spectra in PEDOT, in which the C=C stretching vibration becomes narrow, conclude that this is evidence of a change of resonance in the PEDOT chains from benzoid to a quinoid form [18,36,37,40,41,42].

PEDOT:PSS has two conjugated π-electrons on the C_α_=C_β_ bond in the benzoid structure. Thus the symmetrical C_α_=C_β_ stretching vibration changing from the benzoid to the quinoid structure will show a shift to red [40]. Figure 9b analyzes the position of the C_α_=C_β_ symmetric stretching band near 1434 cm^−1^ for the pristine and post-treated films. As expected, the C_α_=C_β_ symmetric stretching vibration shows a small shift of its band position from 1432 cm^−1^ (pristine and vapor techniques) to 1429 cm^−1^ (drop and dip techniques), meaning a change in the structure conformation from coil (benzoid) to expanded-coil/linear (quinoid) conformation [18]. Quinoid conformation improves conductivity in the treated films by increasing the charge carrier mobility resulting from a rearrangement of chains. This rearrangement is also promoted by the discussed enrichment of PSS in the surface that results in an enriched-PEDOT bulk in the post-treated films. In the case of the vapor post-treated film, such conformational change is not detected by a shift in the band at 1429 cm^−1^ with respect to the pristine film, so we expect that the rearrangement only occurs in a superficial region of the film. These results show a clear correlation between the data found in the electronic characterization and AFM and Raman spectroscopies; the increase of aggressivity of the treatments from vapor to dip techniques results in deeper conformational changes in the bulk from benzoid to quinoid conformation, explaining the increase of conductivity and the increase of roughness in the post-treated films. Thus, a self-assembling process in the nanostructure of PEDOT:PSS can be performed by controlling aggressivity of IPA treatments for application on the hybrid heterojunction.

**Figure 11:**
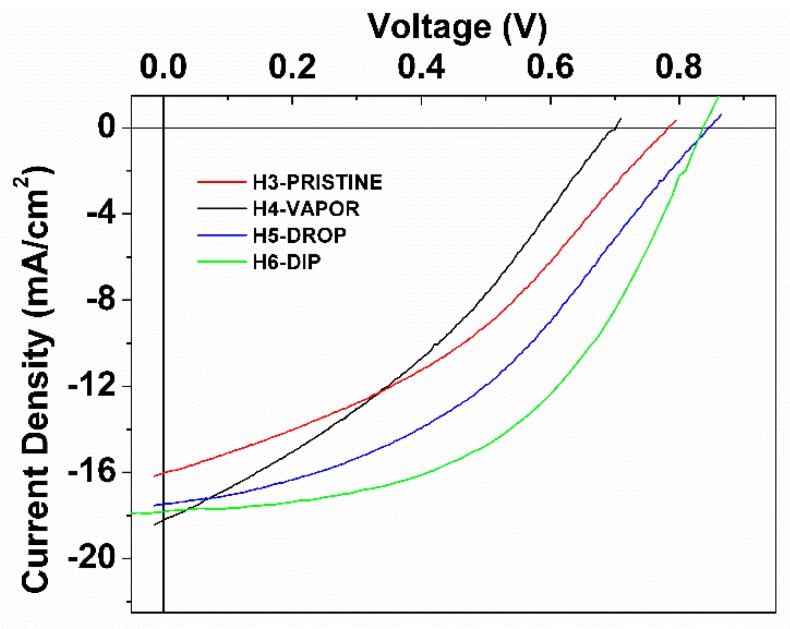
Current density–voltage curves under 100 mW/cm^2^ AM 1.5G illumination for hybrid photovoltaic structures using pristine PEDOT:PSS film post-treated IPA PEDOT:PSS films using VAPOR, DROP, and DIP techniques.

Hybrid pin structures with pristine and post-treated PEDOT:PSS films were fabricated. Figure 11 and Table 3 show the *J*(U) characteristics of the h-pin structures labeled as H3-PRISTINE, H4-VAPOR, H5-DROP, and H6-DIP structures. The H3-PRISTINE reference structure exhibits *J*(U) characteristics as *V*_oc_ = 780 mV, *J*_sc_ = 16 mA/cm^2^, FF = 0.36, and a PCE = 4.6%. The lowest open-circuit voltage of *V*_oc_ = 695 mV was obtained in the H4-VAPOR structure; characterization of post-treated PEDOT:PSS films demonstrated that the effect of post-treatment using the vapor technique on electronic and morphological characteristics is weak and limited to the surface. Moreover, the nature of the vapor treatment may produce nonuniform changes in the surface. This can be confirmed by the variability in work function values obtained for the vapor post-treated film characterization (Table 2) and can explain the low *V*_oc_ in the H4-VAPOR structure. In contrast, the H5-DROP and H6-DIP structures present higher open-circuit voltages than that in H3-PRISTINE structure. In the previous section, it was demonstrated that drop and dip post-treated PEDOT:PSS films were modified by IPA forming an enriched-PEDOT bulk with preferential quinoid conformation and an enriched-PSS surface (Figure 4b). This arrangement in the internal nanostructure of PEDOT:PSS films can improve the electron-blocking process and hole collection. This characteristic can also be enhanced by the increase of work function in the post-treated PEDOT:PSSS layer due to IPA treatments (Figure 4a), explaining the improvement of *V*_oc_ and *J*_sc_ in the H5 and H6 structures.

It is also observed that the changes of conformation from benzoid to quinoid in PEDOT improve charge transport and conductivity, reducing serial resistance in the hybrid structures. This effect is enhanced by the aggressivity of the dip technique. In contrast, low shunt resistance is observed in the H6 structure compared to that in reference a-Si:H structures. A hypothesis is that aggressivity of the dip technique treatment somehow affects the PEDOT:PSS films affecting also a-Si:H layer deposition and reducing shunt resistance. However, using Raman, FTIR, or AFM characterization there is no evidence of degradation of PEDOT:PSS films. Fill factor values of the fabricated structures are shown in Table 2 and Table 3. Low FF (< 45%) values are presented for all structures, including the a-Si:H reference structure. Then, our conclusion is that low FF values are a consequence of the general configuration of the structures and are not completely related to the hybrid frontal heterojunction. In addition, all fabricated hybrid structures using PEDOT:PSS layer showed higher *J*_sc_ (>15 mA/cm^2^) than that in the a-Si:H reference structure.

**Figure 12:**
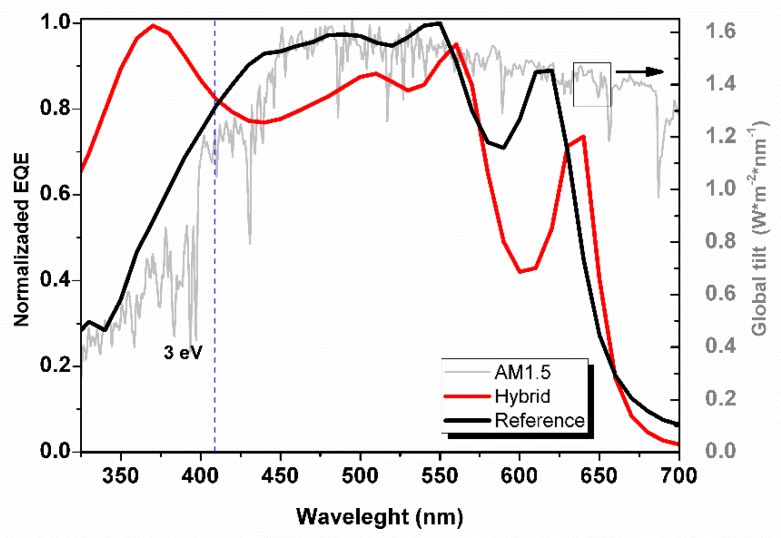
(**a**) External quantum efficiency (EQE) spectra of a photovoltaic structures based on amorphous silicon (a-Si:H) (REFERENCE) and a hybrid photovoltaic structure with a buffer layer and a PEDOT:PSS film treated with DIP in IPA (H6-DIP). (**b**) Comparison of EQE spectra in the range from 300 nm to 450 nm obtained using both LED and GLOBAL source.

Figure 12 shows the normalized external quantum efficiency (EQE) measured for the best hybrid structure (H6-DIP structure) and a a-SI:H reference. There are small differences between the trends of the hybrid and reference structures over the visible–IR range wavelength (420–800 nm) related to photon absorption and charge carrier collection at the bulk. The main difference between spectra in this region is that the reference structure shows an EQE maximum at a wavelength of 450 nm, in contrast to hybrid structures that show a minimum at the same wavelength (450 nm) but a maximum at 524 nm. This behavior has also been observed in similar structures reported by Lee et. al. [13], and seems to be characteristic of PEDOT:PSS/a-Si:H structures. The hybrid structure shows an exceptional response at wavelengths below 420 nm compares to that in reference structure. A clear improvement in the charge carrier collection due to the implementation of the hybrid heterojunction in the frontal interface was found. This improvement is clearly related to the properties of the post-treated PEDOT:PSS and the hybrid heterojunction in the frontal interface as we discussed before: (i) low PEDOT:PSS light absorption (Figure 5), (ii) reduction of electron diffusion by the electron-blocking PSS-enriched layer (Figure 4), and (3) enhancement of the internal electric field due to the buffer layer.

Table 4 compares the performance characteristics of the H6-DIP structure with those reported in the literature for structures based on a PEDOT:PSS/a-Si:H interface [13] and a high-performance amorphous silicon structure fabricated at low temperature (<200 °C) [43,44]. It is worth mentioning that the hybrid structures reported to date reach the highest short-circuit current values *J*_sc_ ~17.83 mA/cm^2^ without using a complex texture process to enhance optical absorption. In contrast, FF values in these structures are lower than those for pure amorphous silicon solar cells. To address this issue, further investigation should be undertaken for optimization of the ITO/PEDOT:PSS interface. However, PCEs of the hybrid structures are comparable to those in a well-optimized amorphous silicon structure fabricated at low deposition temperature [43]. This demonstrates the potential of the PEDOT:PSS to substitute complex frontal interface schemes (p-SiC:H, µm-Si, SiO_x_:H, etc.) that use inorganic materials. From the technological point of view, hybrid heterojunctions have advantages as reduction of vacuum and high-temperature stages and simplification of the optimization of p-type layer creating abrupt interfaces.

Finally, Table 4 presents the comparison between performance characteristics for superstrate-type hybrid and substrate-type hybrid solar cells. The final efficiency of the superstrate-type configuration reported here (PCE = 7.55%) is similar to that in the substrate-type configuration (PCE = 7.40%) [13]. This proves that PEDOT:PSS film is fully compatible with the subsequent PECVD depositions of the a-Si:H layers, and the inferior performance reported in previous studies [13,16,17,24] is attributable to the absence of an optimized hybrid heterojunction and is not caused by degradation of the polymer due to plasma exposure or temperature. Then, it is important to note that the approaches discussed in this work can somehow be extended to other technologies of solar cells (HIT, µ-Si:H, and organic solar cells) in order to improve and simplify the fabrication process.

## 4. Conclusions

We demonstrate here that PEDOT:PSS is an ideal material to substitute p-type inorganic materials for use in frontal interfaces. By tailoring surface of PEDOT:PSS and using a PEDOT:PSS/buffer/a-Si:H heterojunction, the optical transparency of the frontal interface and “built-in” electrical field in an amorphous solar cell were improved. Consequently, higher *J*_sc_ and *V*_oc_ were obtained compared with those in the hybrid structure without a buffer layer. Different IPA post-treatment techniques were used to control a self-assembling process in the nanostructure of the PEDOT:PSS layer, forming a PSS-enriched surface and a PEDOT-enriched bulk without degradation at the molecular level. As a result, the work function and the electrical conductivity of the polymer films were increased. These post-treated PEDOT:PSS films were used in hybrid structures, and the performance characteristics were improved in comparison to those in the amorphous silicon reference.

The best performance characteristics (*J*_sc_ = 17.83 mA/cm^2^, *V*_oc_ = 0.84 V FF = 50% and PCE = 7.55%) were obtained using a PEDOT:PSS/buffer/a-Si:H heterojunction in the hybrid structure fabricated with a PEDOT:PSS layer treated with the dip technique. The increase of *J*_sc_ was explained by a remarkable improvement in charge carrier collection due to the nanostructural arrangement of the PEDOT:PSS layer, the alignment of bands and high transmittance of the hybrid frontal interface. Although the structure is a planar substrate-type configuration and the PEDOT:PSS layer has been exposed to glow discharge, the reached efficiency is the highest reported to date for similar hybrid solar cells and those in textured well-optimized amorphous silicon solar cells fabricated at low deposition temperature (<200 °C).

## Figures and Tables

**Table 1:** Performance parameters of a-Si:H reference structure and hybrid structures with and without a buffer layer.

Sample	Jsc (mA/cm^2^)	Voc (V)	FF (%)	PCE (%)	Rs (Ω cm^2^)	Rsh (Ω cm^2^)
**a-Si:H REFERENCE**	13.02	0.89	45.5	5.27	30	1.4k
**H1- NO_BUFFER**	15.43	0.64	40.0	3.95	22	109
**H2- BUFFER**	19.8	0.86	34.0	5.78	34	100

**Table 2:** Electrical and morphological characteristics of pristine PEDOT:PSS film and post-treated PEDOT:PSS films.

	PEDOT:PSS			
	PRISTINE	VAPOR	DROP	DIP
**Conductivity (S/cm)**	0.54	1.68	20.58	47.88
**Work function (eV)**	4.97–5.02	5.08–5.14	5.01–5.11	5.10
**RMS Roughness (nm)**	1.57	1.80	1.65	1.93
**Surface Skewness (Sk)**	0.551	0.246	0.004	0.056
**Surface Kurtosis (Ku)**	7.74	4.97	3.39	3.26

**Table 3:** Performance parameters of hybrid photovoltaic structures fabricated with pristine and post-treated PEDOT:PSS films.

Sample	Jsc (mA/cm^2^)	Voc (V)	FF (%)	PCE (%)	Rs (Ω cm^2^)	Rsh (Ω cm^2^)
**H3-PRISTINE**	16.02	0.78	36.9	4.61	33	125
**H4-VAPOR**	18.22	0.695	34.0	4.30	30	76
**H5-DROP**	17.48	0.84	40.9	6.0	31	298
**H6-DIP**	17.83	0.84	50.44	7.55	15	303

**Table 4:** Comparison of performance parameters of hybrid photovoltaic structure fabricated in this work and similar structures reported in literature.

Type	Frontal interface	Deposition Temp.	Texturing	Jsc (mA/cm^2^)	Voc (V)	FF (%)	PCE (%)	REF.
**Hybrid** **Superstrate-type**	PEDOT:PSS/buffer SiC:H/a-Si:H	175 °C	NO	17.83	0.84	50.44	7.55	This work
**Hybrid** **Substrate-type**	PEDOT:PSS/doped-buffer/a-Si:H	--	NO	19.1	0.800	48.0	7.40	[13]
**Inorganic** **Superstrate-type**	buffer/p-a-SiOx:H/a-Si:H	175-200 °C	YES	13.35	0.840	75.0	8.40	[43]
